# Early detection of soluble CD27, BTLA, and TIM-3 predicts the development of nosocomial infection in pediatric burn patients

**DOI:** 10.3389/fimmu.2022.940835

**Published:** 2022-07-26

**Authors:** Julia A. Penatzer, Robin Alexander, Shan Simon, Amber Wolfe, Julie Breuer, Josey Hensley, Renata Fabia, Mark Hall, Rajan K. Thakkar

**Affiliations:** ^1^ Center for Clinical and Translation Research, The Research Institute at Nationwide Children’s Hospital, Columbus, OH, United States; ^2^ Biostatistics Resource, The Research Institute at Nationwide Children’s Hospital, Columbus, OH, United States; ^3^ Division of Critical Care Medicine, Nationwide Children’s Hospital, Columbus, OH, United States; ^4^ Department of Pediatric Surgery, Burn Center, Nationwide Children’s Hospital, Columbus, OH, United States

**Keywords:** pediatric thermal injury, soluble proteins, immune checkpoint inhibitors, nosocomial infection, inflammation, immune dysfunction

## Abstract

Thermal injury induces concurrent inflammatory and immune dysfunction, which is associated with adverse clinical outcomes. However, these effects in the pediatric population are less studied and there is no standard method to identify those at risk for developing infections. Our goal was to better understand immune dysfunction and identify soluble protein markers following pediatric thermal injury. Further we wanted to determine which early inflammatory, soluble, or immune function markers are most predictive of the development of nosocomial infections (NI) after burn injury. We performed a prospective observational study at a single American Burn Association-verified Pediatric Burn Center. A total of 94 pediatric burn subjects were enrolled and twenty-three of those subjects developed a NI with a median time to diagnosis of 8 days. Whole blood samples, collected within the first 72 hours after injury, were used to compare various markers of inflammation, immune function, and soluble proteins between those who recovered without developing an infection and those who developed a NI after burn injury. Within the first three days of burn injury, innate and adaptive immune function markers (ex vivo lipopolysaccharide-induced tumor necrosis factor alpha production capacity, and ex vivo phytohemagglutinin-induced interleukin-10 production capacity, respectively) were decreased for those subjects who developed a subsequent NI. Further analysis of soluble protein targets associated with these pathways displayed significant increases in soluble CD27, BTLA, and TIM-3 for those who developed a NI. Our findings indicate that suppression of both the innate and adaptive immune function occurs concurrently within the first 72 hours following pediatric thermal injury. At the same time, subjects who developed NI have increased soluble protein biomarkers. Soluble CD27, BTLA, and TIM-3 were highly predictive of the development of subsequent infectious complications. This study identifies early soluble protein makers that are predictive of infection in pediatric burn subjects. These findings should inform future immunomodulatory therapeutic studies.

## Introduction

Thermal injuries are a public health crisis, affecting approximately 2 million people in the United States annually, with roughly half of these occurring in children (1). Despite continued improvements in burn care, which have reduced mortality, infection-related complications remain high (2–4). The American Burn Association states the most clinically relevant complications for pediatric burn patients are pneumonia, urinary tract infection, cellulitis, bacteremia, wound infection, and respiratory failure (5). In fact, over 60% of patients with large burns will develop an infection (6, 7).

Pediatric burn injury has been shown to lead to a dysregulated inflammatory response releasing both pro- (e.g., interleukin (IL)-1, -6) and anti-inflammatory (e.g., IL-4, -10) cytokines, but at differing concentrations than adult burn patients (8–10). The excessive and concurrent release of pro- and anti-inflammatory cytokines (i.e., IL-6, IL-10) that occurs over a short period of time can lead to altered immune function, further resulting in compromised function of multiple organ systems, and ultimately leading to infectious complications or mortality (9–12). Clinicians also face an extreme challenge as this profound inflammatory response that burn patients display closely resembles the excessive and concurrent pro- and anti-inflammatory cytokine release seen in patients who present with infections/sepsis (13). Additionally, burn patients often undergo frequent surgical procedures which again stimulates the inflammatory cascade and augment physiologic variables such as heart rate, blood pressure and temperature. The additional release of cytokines as well as burn induced physiologic changes can mirror signs of infection, again making the diagnosis of infection/sepsis more challenging. While sepsis guidelines typically encourage early source control and anti-microbial therapy, its overuse has significant consequences such as the development of antibiotic resistance organisms. Therefore, studies have looked at other markers of the innate and adaptive immune system to better identify those at risk. Both innate and adaptive immune cells (e.g., macrophages and lymphocytes, respectively) have shown to be decreased following adult burn injury and further suppressed in those who develop infections (6, 14). We have previously shown markers of both innate and adaptive immune *function* are also decreased in pediatric burn patients who develop infections (7, 12). However, there is no standard method to identify those at risk, and the underlying mechanisms and the sequence of events that lead to immunosuppression in burn patients are still largely unknown. We hypothesize this suppression in pediatric burn patients is associated with the early release of soluble protein markers, that have a critical role in immune regulation, and these markers may serve as a risk factor for the subsequent development of NI.

## Methods

This study was conducted at Nationwide Children’s Hospital, a free-standing, quaternary care, American Burn Association-verified Pediatric Burn Center in Columbus, Ohio. The study was approved by the local Institutional Review Board and informed consent (and assent when appropriate) was obtained from subjects’ legal guardians prior to study participation (IRB15-00995). Inclusion criteria included hospitalization due to acute burn injury, age < 18 years, and hospital stay that was expected to last at least 3 days. Exclusion criteria included known immunodeficiency and current use of systemic immunosuppressive medications. Healthy control (HC) subjects were enrolled from the outpatient phlebotomy laboratory on a volunteer basis with informed consent (and assent when appropriate) obtained from the subject’s legal guardians. Patients were enrolled prospectively into this observational study between 2016 and 2021. A total of 121 pediatric burn patients were enrolled, of which 94 subjects had a whole blood sample obtained within the first 72 hours of injury and were used for subsequent analysis. Whole blood samples from HC subjects were also obtained. Healthy subjects were excluded if they met any of the following criteria: recent or current fever, history of chronic inflammatory disease, history of transplantation, current use of antibiotics or systemic immunosuppressive medications, or history of systemic corticosteroid use within the past month. A total of 17 HC subjects, who showed no significant age difference, were included in the analysis.

### Immune function testing

Within 1 h of collection, 50 µL of heparinized whole blood was added to two tubes containing highly standardized lipopolysaccharide (LPS) stimulation reagent (500 pg/mL LPS from Salmonella abortus equi, Enzo Life Sciences, Inc., Farmingdale, NY) and incubated for 4 h at 37°C. Fifty µL of heparinized whole blood was also added to two tubes containing highly standardized phytohemagglutinin (PHA) stimulation reagent (Sigma Aldrich, St. Louis, MO) and incubated for 24 hours at 37°C. After incubation, the samples were centrifuged at 850 X g for 5 min. The supernatants were collected and stored at −80°C for batch analysis. Tumor necrosis factor alpha (TNFα) production capacity from LPS stimulation was quantified using the Immulite 1000 automated chemiluminometer (Siemens Healthcare Diagnostics, Deerfield, IL). The quantification of LPS stimulated TNFα production on the Immulite 1000 is used as a standardized assay for several multi-center research studies, within the Immune Surveillance Laboratory at the Research Institute at Nationwide Children’s Hospital, in evaluating pediatric critical illness (e.g., burns, trauma, sepsis) and is used to ensure rigor reproducibility. However, due to limited sample volume from our pediatric patients, multiplex kits for the Bio-Plex 200 system (BioRad, Hercules, CA, USA) were used for all other cytokine assays. Specifically, IL-4, IL-10, and interferon gamma (IFNγ) production from PHA stimulation was measured by multiplex assay and quantified using the Bio-Plex 200, according to the manufacturer’s instructions. Stimulation assays were performed in duplicate for each blood sample and values reported represent the average value from each set of duplicates. The lowest values were used for analysis in patients who had more than one sample obtained within the first 72 hours. LPS and PHA stimulation solution was manufactured bimonthly in the Immune Surveillance Laboratory at the Research Institute at Nationwide Children’s Hospital and quality controlled to ensure the intra-batch coefficient of variation from healthy donor replicates was <10% for TNFα and IL-10 response, respectively. LPS-stimulated TNFα and PHA-stimulated IL-4, IL-10, and IFNγ were chosen based on our previous studies indicating their prediction of NI in pediatric burn patients (7, 12).

### Flow cytometry

Whole blood samples for flow cytometry were collected in EDTA tubes (Becton Dickinson, Franklin Lakes, NJ). Absolute CD4+ lymphocyte counts, percent programmed death ligand-1 (PD-1) expression on CD4+ cells (CD4+CD279+), percent cytotoxic T-lymphocyte antigen-4 (CTLA-4) expression on CD4+ cells (CD4+CD28+CD152+), CD4+CD25+CD127lo regulatory T cells (Tregs), absolute CD66b+ neutrophil counts, absolute CD14+ monocyte counts, CD14+ monocyte HLA-DR expression, and natural killer (NK) cells were determined by flow cytometry. Briefly, 100 μL of whole blood was incubated with human IgG (Invitrogen, Carlsbad, CA) for 10 min at 4°C. Each sample was then stained with the appropriate fluorochrome-conjugated specific antibodies for 30 minutes at room temperature. The following antibodies (BD Biosciences, San Jose, CA or Biolegend, San Diego, CA) were used: FITC CD66b (clone G10F5; Biolegend), V450 CD14 (clone M0P9; BD), BV605 human leukocyte antigen (HLA)-DR (clone G46-6; BD),V450 CD3 (clone UCHT1; BD), BV711 CD4 (clone SK3; BD), BV605 CD8 (clone SK1; Biolegend), APC CD25 (clone M-A251; Biolegend), APC CD279 (PD-1, clone MIH4; BD), APC CD152 (CTLA-4, clone L3D10; Biolegend), PE CD127 (HIL-7R-M21; BD), PE CD28 (clone CD28.2; Biolegend), and FITC CD56 (clone HCD56; Biolegend). The samples were lysed (BD FACS TM Lysing Solution, BD Biosciences, San Jose, CA) for 15 minutes at room temperature. Following centrifugation, cells were washed and fixed with 4% paraformaldehyde (Cytofix, BD Biosciences, San Jose, CA) for 30 minutes at 4°C. The cells were again washed, centrifuged, and resuspended in stain buffer (BSA, BD Biosciences). The samples were processed using a LSRII cytometer and all data was analyzed with FlowJo (FLOWJO, LLC Data Analysis Software).

For innate cells, the gating strategy included initial doublet exclusion followed by granulocyte exclusion and gating of neutrophils (FITC CD66b+). Of the remaining cells, CD14+ cells were selected, and within that population %HLA-DR was determined relative to the fluorescence minus one control. For adaptive immune cells, the gating strategy included initial doublet exclusion, followed by CD3+ and CD3- cells gating with V450. Further gating of the CD3+ T-cells around CD4+ and CD8+ T-cells was done using BV711 (CD4+ cells) and BV605 (CD8+ cells), respectively. Tregs were gated from the CD4+ T cell population using APC CD25 and PE CD127 (CD4+CD25+CD127lo). Additionally, within the CD4+ T cell population, %PD-1 and %CTLA-4 were determined using APC CD279 (CD4+CD279+) or APC CD152 and PE CD28 (CD4+CD28+CD152+), respectively and fluorescence minus one controls. Gating of CD3- cells around NK cells was performed using FITC CD56. Both experiments were performed in duplicate, and the mean (cells/μL) was analyzed. In patients who had more than one sample obtained within the first 72 hours the lowest values were used for analysis. CountBright™ absolute counting beads (Life Technologies, Carlsbad, CA) were used to determine absolute cell counts.

### Plasma measurements

Whole blood samples for unstimulated plasma analyses were collected in heparin tubes (Becton Dickinson). Within 1 h of collection, tubes were centrifuged at 1000 X g for 5 min and plasma was collected and stored at -80°C for subsequent analysis. For cytokine analysis, samples were assayed using the Bio-Plex multiplexed magnetic bead-based immunoassay reagent kit for quantification of the following targets: IL-2, IL-6, IL-8, IL-10, IL-12, and IL-17 (Bio-Rad, Hercules, CA). Samples were analyzed on the Bio-Plex 200 System platform, according to the manufacturer’s instructions. Additionally, soluble protein targets that play a role in the regulation of T cells were assayed and quantified using the Invitrogen Immuno-Oncology Checkpoint 14-Plex Human multiplex for the following targets: BTLA, CD137, CD152, CD27, CD28, CD80, GITR, HVEM, IDO, LAG-3, PD-1, PD-L1, PD-L2, and TIM-3 (Thermo Fisher Scientific, Waltham, MA). Samples were analyzed on the Bio-Plex 200 System platform, according to the manufacturer’s instructions. Cytokine and soluble protein assays were performed in duplicate for each blood sample and values reported represent the average value from each set of duplicates. In patients who had more than one sample obtained within the first 72 hours the highest values were used for analysis.

### Demographics

The following patient information was collected from the electronic medical record: age, sex, mechanism of burn injury, presence of inhalational injury and percent total body surface area (TBSA) burn (**Table 1**).

**Table 1:** Demographics.

Variable	Burn Without NIN=71	Burns With NIN=23	HC N=17	No NI vs NIp-value	No NI vs HCp-value	NI vs HCp-value
**Age, y**	4.2 [1.9-9.0]	4.3 [2.0-11.0]	9.8 [3.7-15.0]	>0.99^a^	0.18^a^	0.47^a^
**Male Gender (%)**	63	65	53	0.58^b^	>0.99^b^	0.52^b^
**Flame Injury (%)**	39	61	NA	0.09^b^	–	–
**Inhalational Injury (%)**	0	17	NA	**0.0029^b^**	–	–
**TBSA Burn (%)**	10 [7-13]	26 [16-46]	NA	**<0.0001^c^**	–	–

Data are median with interquartile range. ^a^ comparison of groups using Kruskal-Wallis with Dunn’s correction; ^b^ comparison of groups using Fisher’s Exact test; ^c^ comparison of groups using Wilcoxon rank sum test. Boldface type indicates statistical significance. NI, nosocomial infection; HC, healthy controls; NA, not available; TBSA, total body surface area.

### Infection data

NIs were identified through prospective review of the electronic medical record and were defined as new infections that were identified within 30 days from hospital admission according to the Centers for Disease Control criteria (N=23; **Table 2**) (15). Infection testing was obtained using our burn centers sepsis guidelines as well as at the discretion of the treating clinicians. Diagnosis of infection was confirmed independently by two surgeons who were blinded to the immune function testing results.

**Table 2:** Infectious complications.

Subject ID	Organism	Source	Post-Burn Day Diagnosed
1	MRSA	Urine	13
8	Streptococcus Pneumoniae	Lung	4
18	Streptococcus Pneumoniae	Lung	3
19	Streptococcus Pneumoniae and Haemophilus Influenzae	Lung	6
24	MRSA	Blood	9
25	Bacteremia	Blood	4
26	MRSA and Streptococcus Pneumoniae	Lung	6
27	MRSA and Group B Beta-Hemolytic Streptococcus	Lung	11
31	Hospital Acquired Pneumonia	Lung	10
36	Pseudomonas Aeruginosa	Blood	15
37	Group A Streptococcus	Lung	3
45	Herpes Simplex Virus	Blood	8
54	Influenza A	Lung	19
63	Coagulase Negative Staphylococcus Aureus	Urine and Lung	3
85	Staphylococcus Aureus Moraxella	Burn Wound and Lung	11
92	Enterobacter cloacae	Burn Wound and Lung	5
99	Enterobacter cloacae	Lung	10
102	Staphylococcus Aureus Enterococcus casseliflavus	Burn Wound	6
103	Staphylococcus Aureus	Lung	3
104	Staphylococcus Aureus	Burn Wound	4
109	Staphylococcus Aureus	Urine	12
110	Staphylococcus Aureus	Burn Wound	8
112	Pneumonia with Moraxella	Lung	12

MRSA, Methicillin Resistant Staphylococcus Aureus.

### Statistical analysis

Descriptive statistics were summarized using the median (inter-quartile range) for continuous variables and frequency (percentage) for categorical variables. Fisher’s exact test was conducted to determine statistically significant differences between groups for categorical variables. A Kruskal-Wallis was conducted to compare differences in demographic continuous variables. The Wilcoxon rank sum test was used to determine if there were differences in continuous variables between the groups when there were only two possible groups (burns without NI and burns with NI) or Kruskal-Wallis test was conducted to determine if there were differences in continuous variables between the groups when there were three groups (burns without NI, burns with NI and HC). For comparisons between three groups, Dunn’s multiple comparison test with Bonferroni’s multiple comparisons correction for continuous variables and Bonferroni’s correction for multiple comparisons with categorical variables were used. The performance of immune functions for diagnosis of infection were analyzed using receiver operating characteristic curves (ROC). Youden’s index was used to determine the optimal cut point for each marker of immune function by maximizing the sum of the specificity and sensitivity. Hypothesis testing was conducted at a 5% type I error rate (α=0.05). All analyses were conducted in R version 4 (R Studio, Boston, MA) and Prism 9 software (GraphPad, Inc, San Diego, CA) (16–22).

## Results

Ninety-four thermally injured subjects were included in the present study. All 94 subjects (71 with No NI; 23 with NI) had at least one blood sample obtained within the first 72 hours after injury. Seventeen healthy subjects were also enrolled in this study and were not significantly different in age from the burn cohorts. Of the burn subjects, 23 developed a NI with a median time to diagnosis of 8 days (range: 3 to 19 days post injury). No deaths occurred within this cohort. Subjects who developed NI had larger percent TBSA burn (26% *vs*. 10%; p<0.0001) and were more likely to have inhalational injury (4 patients [17%] *vs*. 0 patients [0%]; p=0.0029), but their demographics were otherwise not statistically significant from those without NI (No NI; **Table 1**). Infection sites and identified pathogens for subjects with NI are shown in **Table 2**. In the present study, the most common infection for the subjects occurred in the lower airway (61%) followed by burn wound infections (22%). Four subjects were noted to have blood stream infections: three subjects with bacteremia and one with herpes simplex viremia. Two subjects had urine infections.

### Unstimulated plasma cytokine production

**Figure 1:**
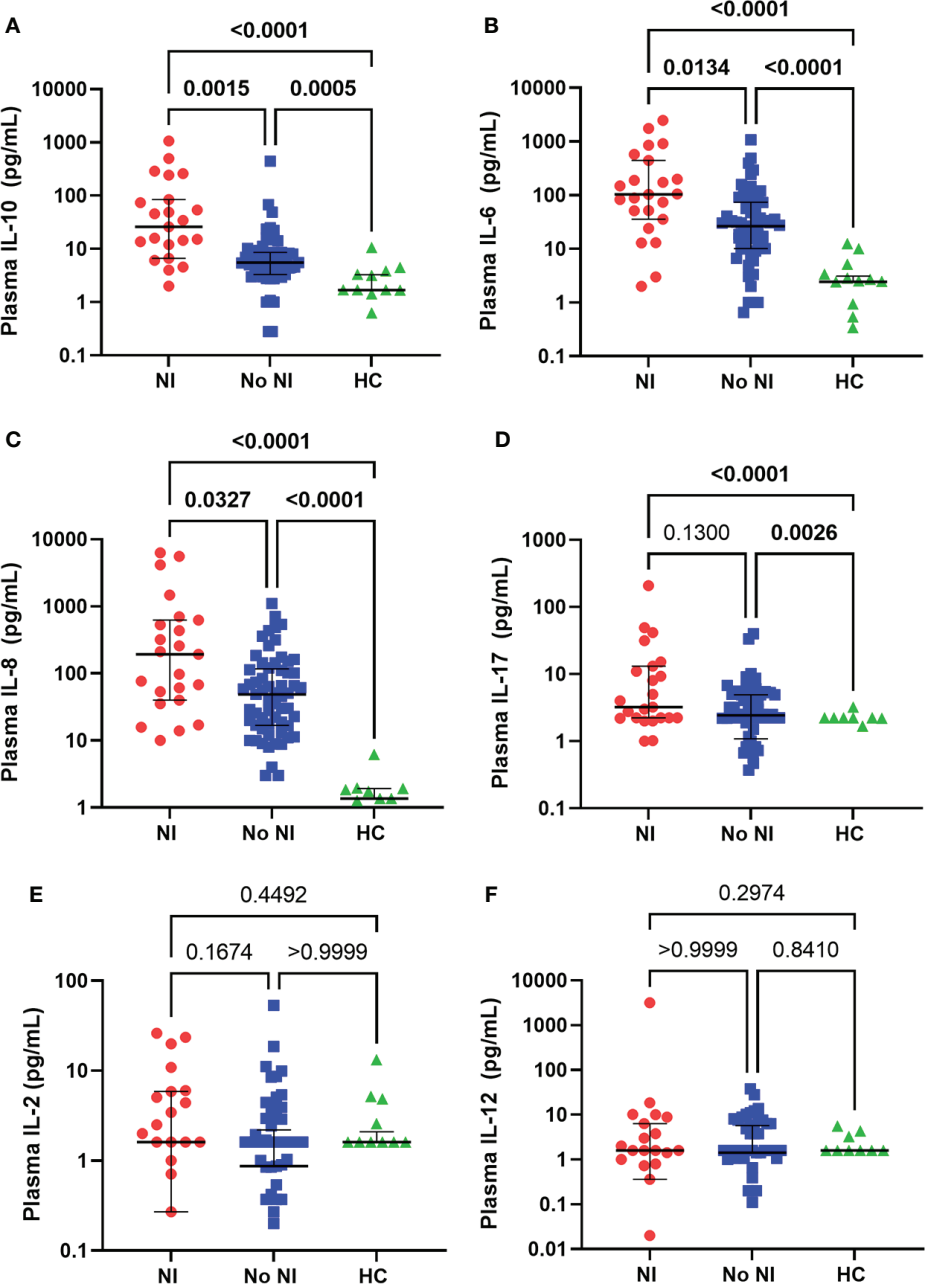
Plasma cytokines levels within the first 72 hours following pediatric thermal injury. Burn injury, regardless of infection (NI, n=23 and No NI, n=61) significantly decreased median plasma levels of IL-10 **(A)**, IL-6 **(B)**, IL-8 **(C)**, and IL-17 **(D)** compared to healthy controls (n=17, except IL-8 n=11). Subjects who went on to develop NI had higher plasma levels of IL-10 **(A)**, IL-6 **(B)**, and IL-8 **(C)** compared to those who recovered from burn injury without developing an infection. There were no significant differences in the plasma levels of IL-2 **(E)** and IL-12 **(F)** between groups in the first 72 hours. Statistical analysis was performed using one-way ANOVA plus Dunn’s test. Lines represent median and interquartile range; dots represent the individual data points. NI, nosocomial infection (red); No NI, no infection (blue); HC, healthy control (green).

In the unstimulated plasma samples obtained within the first 72 hours of injury, both burn patients who developed NI and burn patients who recovered without developing NI had significantly elevated median levels of IL-10 (NI: 25.92 pg/ml; No NI: 5.53 pg/mL), IL-6 (NI: 103.6 pg/mL; No NI: 26.58 pg/mL), IL-8 (NI: 193 pg/mL; No NI: 48.92 pg/mL), and IL-17 (NI: 3.230 pg/mL; No NI: 2.430 pg/mL) compared to HCs (IL-10: 1.69 pg/mL; IL-6: 2.43 pg/mL; IL-8: 1.360 pg/mL; IL-17: 0 pg/mL) (**Figures 1A–D**). Moreover, patients who went on to develop a NI had significantly higher median concentrations of IL-10, IL-6, and IL-8 as opposed to No NI (IL-10: 25.92 *vs*. 5.53 pg/mL; IL-6: 103.6 *vs*. 26.58 pg/mL; IL-8: 193.0 *vs*. 48.92 pg/mL) (**Figures 1A–C**). We did not detect any significant difference in the plasma levels of IL-2 and -12 between subjects in the first 72 hours (**Figures 1E, F**). These results suggest a dysregulated cytokine response occurs within the first 3 days after thermal injury and is augmented in those who develop NI.

### Immune cell presence

**Figure 2:**
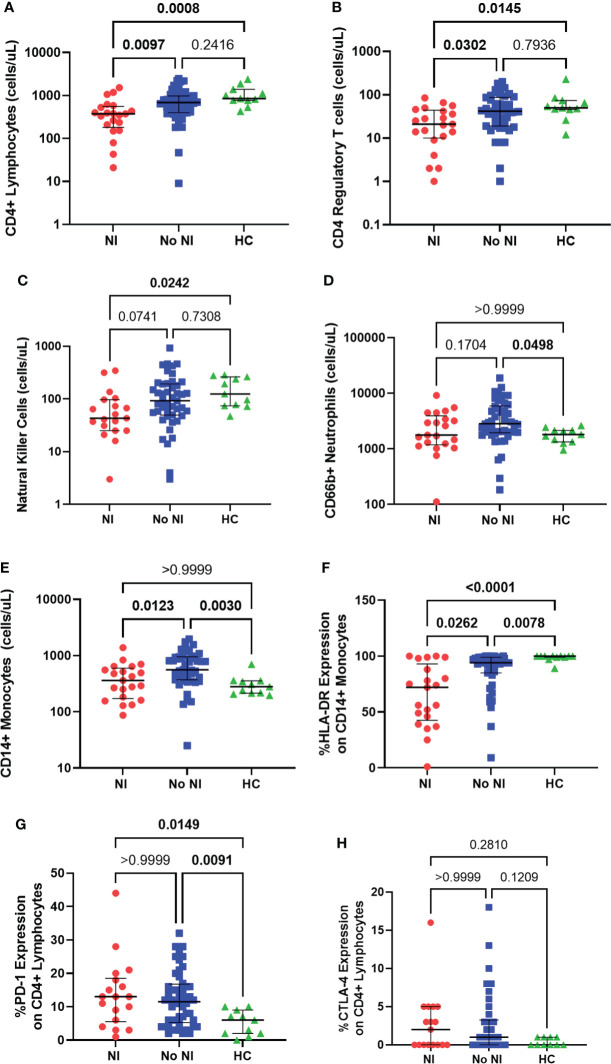
Immune cell presence within the first 72 hours following pediatric thermal injury. Adaptive immune cells as measured through absolute CD4+ lymphocytes **(A)**, CD4 regulatory T cells **(B)** and natural killer cells **(C)** were decreased in subjects who went on to develop an infection (n=21, 21, 19, respectively) relative to those who did not (n=48, 47, 46, respectively) and healthy controls (n=11). Innate immune cells as measured through CD66b+ neutrophils **(D)** and CD14+ monocytes **(E)** were elevated in children who recovered without developing an infection (n=46, 47, respectively) relative to healthy controls (n=11) and those who did develop an infection (n=21). CD14+ monocyte HLA-DR expression **(F)** was significantly lower in children that went on to develop NI (n=21) compared to those patients that did not (n=46) and healthy controls (n=11). Burn injury regardless of infection (NI, n=18 and No NI, n=48) significantly decreased median percent PD-1 expression on CD4+ lymphocytes **(G)** relative to healthy controls (n=11) but displayed no difference between burn groups. There was no significant difference in the percent CTLA-4 expression **(H)** between groups (NI, n=17, No NI, n=42, HC, n=10). Statistical analysis was performed using one-way ANOVA plus Dunn’s test. Lines represent median and interquartile range; dots represent the individual data points. NI, nosocomial infection (red); No NI, no infection (blue); HC, healthy control (green).

We evaluated both adaptive and innate immune cells, using flow cytometry, in burn subjects, within the first 72 hours after injury, and HCs. Burn subjects who developed NI had significantly lower absolute CD4+ lymphocyte counts (375 cells/μL) relative to those who recovered without developing NI (687.5 cells/μL) and HCs (855 cells/μL) (**Figure 2A**). Tregs (CD4+CD25+CD127lo) were also found to be significantly decreased in those who that developed an infection (21.0 cells/μL) versus those that did not (42.0 cells/μL) and HCs (50.0 cells/μL) (**Figure 2B**). NK cells were decreased for those that developed NI (43 cells/μL) from those that did not (92.5 cells/μL) and controls (124 cells/μL) (**Figure 2C**).

Thermally injured children who recovered without developing NI had significantly elevated absolute neutrophil counts (CD66b+; 2821 cells/μL) from controls (1800 cells/μL), but we did not detect any difference, in the first 72 hours, with those that went on to develop a NI (1767 cells/μL) (**Figure 2D**). Absolute CD14+ monocyte counts were also significantly elevated for subjects who recovered without developing NI (560 cells/μL) in the first 72 hours after injury compared to burn subjects who did develop NI (361 cells/μL) and HCs (280 cells/μL); however, there was no significant difference between HCs and those that developed NI (**Figure 2E**). Conversely, %HLA-DR expression on CD14+ monocytes was significantly lower in the subjects who developed NI compared to No NI (72% *vs* 94%) and compared to controls (100%) (**Figure 2F**). Burn injury resulted in significantly increased percent expression of PD-1 on CD4+ lymphocytes (NI: 13%; No NI: 11.50%) relative to HCs (6%) (**Figure 2G**). Similarly, burn subjects had slightly increased percent CTLA-4 expression on CD4+ lymphocytes (NI: 2%; No NI: 1%) relative to HCs (0%) although not statistically significant (**Figure 2H**). Neither percent PD-1 nor CTLA-4 expression displayed any differences between burn groups. These results indicate reduced innate and adaptive immune cells within the first 72 hours after burn injury in those individuals who go on to develop NI relative to their burn counterparts. Therefore, these results suggest that the subsequent infections are likely due to an inappropriate immune cell response; however, it does not appear to be a result of the common inhibitory checkpoint regulators PD-1 and CTLA-4.

### Immune function testing

**Figure 3:**
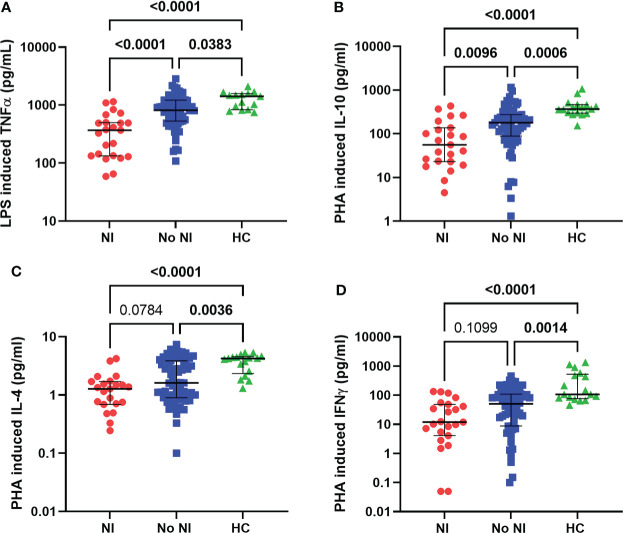
Immune function within the first 72 hours following pediatric thermal injury. Innate and adaptive immune function were measured by ex vivo LPS and PHA stimulation, respectively. Within the first 72 hours of injury, LPS induced TNFα **(A)** was decreased for all burn subjects (NI, n=23 and No NI, n=66) compared to healthy controls (n=15). PHA-induced cytokine-production capacities **(B–D)** were decreased for all burn subjects (NI, n=23 and No NI, n=67) compared to healthy controls (n=17). Subjects who went on to develop an infection had significantly lower LPS induced TNFα **(A)** and PHA induced IL-10 **(B)** compared to those who recovered from burn injury without developing an infection. Statistical analysis was performed using one-way ANOVA plus Dunn’s test. Lines represent median and interquartile range; dots represent the individual data points. NI, nosocomial infection (red); No NI, no infection (blue); HC, healthy control (green).

Innate and adaptive immune function were tested using LPS and PHA stimulations, respectively. In the presence of LPS, healthy monocytes should produce TNFα robustly, while PHA stimulates lymphocyte production of cytokines. Within the first 72 hours, subjects who went on to develop NI had significantly lower TNFα production capacity from LPS stimulation (369 pg/mL) compared to those who recovered without developing NI (817.5 pg/mL) and HCs (1416 pg/mL) (**Figure 3A**). Interestingly, both burn populations (NI and No NI) displayed a significant decrease in PHA stimulated cytokine production relative to HC for all the tested cytokines in the first 3 days (**Figures 3B–D**). However, only PHA-induced IL-10 production capacity was significantly lower in burn subjects who developed NI as compared to No NI (55.57 pg/mL *vs* 178.7 pg/mL) (**Figure 3B**). The findings here suggest that, in addition to having lower systemic immune cells, pediatric burn subjects, who go one to develop infections, have reduced immune function leaving them highly susceptible to infectious complications.

### Unstimulated plasma soluble protein

**Figure 4:**
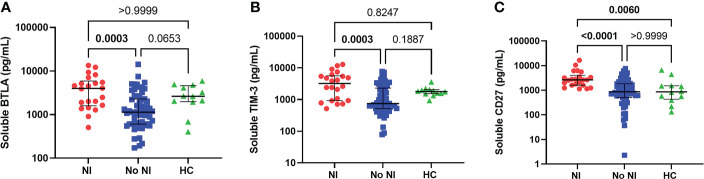
Soluble protein in unstimulated plasma within the first 72 hours following pediatric thermal injury. Soluble BTLA **(A)**, TIM-3 **(B)**, and CD27 **(C)** were all significantly higher in children that went on to develop nosocomial infection (n=22) compared to those patients that did not (n=59) within the first 72 hours after injury. Soluble CD27 **(C)** was also significantly higher from healthy controls (n=12). Statistical analysis was performed using one-way ANOVA plus Dunn’s test. Lines represent median and interquartile range; dots represent the individual data points. NI, nosocomial infection (red); No NI, no infection (blue); HC, healthy control (n=12).

As a follow-up to these observations, we chose to measure soluble proteins, that regulate T cells, from the unstimulated plasma between those that developed NI and those that did not. Soluble BTLA and TIM-3 were significantly increased for those who developed an infection after thermal injury from those that did not (BTLA: 3998 pg/mL *vs* 1142 pg/mL; TIM-3: 3225 pg/mL *vs* 752.1 pg/mL) (**Figures 4A, B**). Interestingly, soluble CD27 was significantly increased, in the first 72 hours, for NI subjects relative to No NI (2652 pg/mL *vs* 872.3 pg/mL) and HC (861.3 pg/mL) (**Figure 4C**). There were no statistically significant differences between the burn subjects or healthy controls for the remaining markers: CD152, CD137, CD28, CD80, IDO, LAG-3, PD-1, PD-L2 (**Supplemental Figure 1**). HVEM, GITR, and PD-L1 were below the limit of detection for at least half of the subjects (median No NI: 0 pg/mL) and were not included in the analysis (data not shown). These findings suggest that elevations in BTLA, TIM-3, and CD27 could be inhibiting an appropriate T cell response after burn injury which ultimately leads to the development of an infection.

### Receiver operating characteristic curves

**Figure 5:**
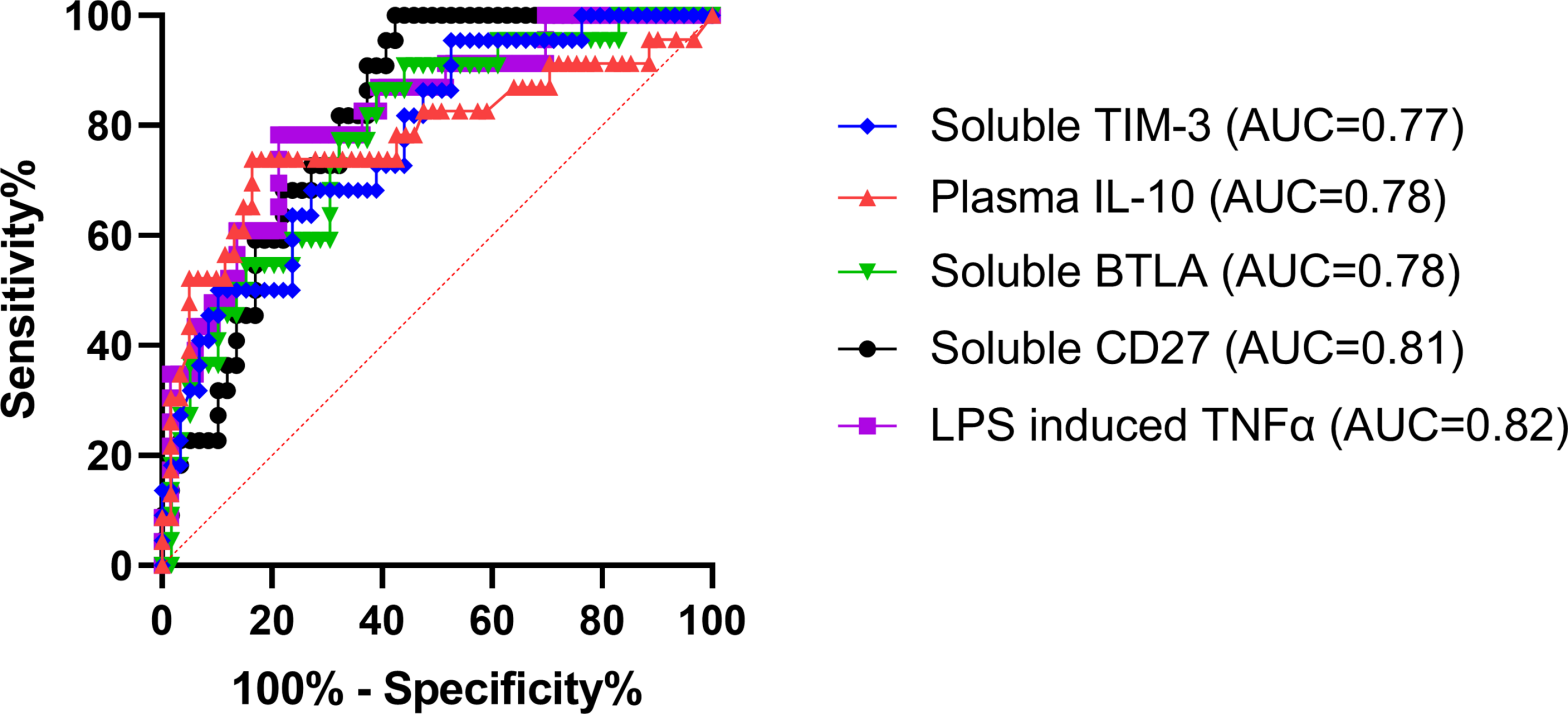
Receiver operating characteristics curve analysis within the first 72 hours following pediatric thermal injury. The area under the receiver operating characteristic curves was highest for LPS-induced TNFα production capacity (0.82; purple) and soluble CD27 (0.81; black). This was followed by soluble BTLA (0.78; green), unstimulated plasma IL-10 (0.78; red), and soluble TIM-3 (0.77; blue). The remaining markers (CD4+, Tregs, CD14+, %HLA-DR expression, plasma IL-6, plasma IL-8, and PHA IL-10) had an AUC ≤ 0.75 (**Supplemental Table 1**). The optimal threshold for the prediction of the development of a nosocomial infection was for <505.8 pg/mL for LPS-induced TNFα, >1129 pg/mL for soluble CD27, >1353 pg/mL for soluble BTLA, and >705.3 pg/mL for soluble TIM-3, and >11.59 pg/mL for plasma IL-10.

ROCs were used to determine the ability of each assay (that displayed a significant difference between burn groups) to predict the subsequent development of NI. Area under the curves (AUCs) were highest for LPS-induced TNFα production capacity (0.82; p<0.0001) and soluble CD27 (AUC=0.81; p<0.0001) (**Figure 5**). This was followed by soluble BTLA (0.78; p<0.0001), unstimulated plasma IL-10 (0.78; p<0.0001), and soluble TIM-3 (0.77; p=0.0002) (**Figure 5**). The remaining markers (CD4+, Tregs, CD14+, %HLA-DR expression, plasma IL-6, plasma IL-8, and PHA IL-10) had an AUC ≤ 0.75 (**Supplemental Table 1**).

The optimal threshold for the LPS-induced TNFα response to predict the development of a NI was < 505.8 pg/mL (sensitivity, 78.26%; specificity, 78.79%). The threshold for soluble plasma protein levels of BTLA, CD27, and TIM-3 were >1353 pg/mL (sensitivity, 86.36%; specificity, 61.02%), >1129 pg/mL (sensitivity, 100%; specificity, 57.63%), and >705.3 pg/mL (sensitivity, 95.45%; specificity, 47.46%), respectively. The threshold for unstimulated plasma IL-10 was >11.59 pg/mL (sensitivity, 73.91%; specificity, 83.61%). These results suggest that LPS-induced TNFα response < 505.8 pg/mL or soluble CD27 concentrations >1129 pg/mL are the best predictors of whether an individual pediatric patient will develop an infection after burn injury.

## Discussion

Pediatric burn injury is a major epidemiological problem with nearly 300 children seen each day in emergency rooms in the United States (23, 24). Advances in the management of these injuries has decreased mortality rates to less than 3%, but infections are still the most common acute complication in patients with significant burn injury (24, 25). As the cellular elements of the immune system are essential to defend against these infections, severe abnormalities have been associated with the development of NI (7, 12, 26, 27). Previous work has identified a number of physiological and metabolic responses to pediatric burn injury and primarily focused on inflammatory responses measuring plasma cytokine profiles (6, 10, 28, 29). However, these heightened inflammatory responses after burn injury, which often resemble the signs of sepsis, occur regardless of infection (13). The findings from our current study are significant as we demonstrate elevated soluble immune proteins at the same time as immune suppression, within the first 72 hours after pediatric thermal injury, in individuals who will develop an infection (median time to diagnosis was 8 days). These soluble proteins point to dysregulation of an appropriate T cell response and were highly predictive of infectious complications. This information with the concurrent immune suppression sheds light onto these dysfunctional biological pathways and may provide useful biomarkers to identify those at risk for infection as well as guide future immunomodulatory therapies.

It is well known burn injury instantaneously causes inflammation and elicits damage associated molecular proteins to stimulate immune cells (30, 31). Typically, after injury there is first a significant release of pro-inflammatory cytokines followed by a delayed anti-inflammatory cytokine response; together they work to counterbalance each other to maintain homeostasis. However, our previous work and this current study show a similar trend to Finnerty et al. (10), indicating elevated levels of both pro-inflammatory (IL-6 and IL-8) and anti-inflammatory (IL-10) cytokines within the first couple days after pediatric thermal injury and are further elevated in patients who go on to develop an infection (**Figures 1A–C**) (7, 10, 12, 32). This dysregulated inflammatory state can alter immune function ultimately leading to infectious complications (10, 12, 32–34).

After the initial inflammatory release, innate immune cells, generally thought of as neutrophils and monocytes, are the first to the wound site. Several adult burn studies have pointed to decreased neutrophil and monocyte counts and decreased cellular function after thermal injury (e.g., phagocytosis, bactericidal activity), and has been suggested to be predictors of sepsis (30, 35–38). We have previously observed similar results to those shown in this study, displaying an inability for those who develop NI to increase their number of absolute CD14+ monocytes unlike those who recovered without an infection (**Figure 2E**) (7). There was, however, no difference between those who developed a NI and HCs. The present study also examined the role of absolute CD66b+ neutrophils but we observed no differences, systemically, between those that would develop a NI and those who recovered without developing an infection or HC (**Figure 2D**). Therefore, pediatric burns may elicit a different cellular response of the innate immune system for absolute neutrophils and monocytes than adults.

Conversely, measurements of innate immune function displayed several distinct decreases following burn injury with further depressed responses in those that go on to develop an infection. Both LPS stimulation and HLA-DR, a major histocompatibility complex II cell surface receptor, are commonly used as markers of immunosuppression (7, 14, 39–43). LPS can influence T cell activation by releasing inflammatory cytokines (e.g., TNFα) on healthy antigen presenting cells. However, decreased LPS-stimulated cytokine production is associated with an increased risk for infection and mortality in both adult and pediatric populations (7, 26, 44–46). Similarly, HLA-DR is commonly found to be decreased following burn injury in both adults and pediatrics and has been linked to development of infections (7, 47, 48). In the present study, we confirm that both burn injury groups (NI and No NI) have significantly decreased %HLA-DR expression and LPS-induced TNFα production relative to HC (**Figures 2F**, **3A**). Further, those patients who go on to develop a NI were significantly lower than that of their burn counterparts. Interestingly, there is a hypothesized link to increased anti-inflammatory cytokines (e.g., IL-10) inhibiting antigen presentation and therefore decreasing ex vivo LPS-stimulated TNF-α production (7, 27, 47, 49, 50). Thus, the NI cohort may be more susceptible to infections as their increased circulating IL-10 inhibits antigen presentation preventing stimulation of T cell immunity.

In this study we also aimed to expand on our previous work observing adaptive immune cells (i.e., lymphocytes) and their function after thermal injury. We have previously shown decreased lymphocyte populations in pediatric burn patients within the first 3 days after injury, and further decreases in those who develop infections (11, 12, 40, 51, 52). In the present study, we also observed NK cells, which are generally thought of as a part of the innate immune response but share lineage with T lymphocytes (34). NK cells are one of the first lines of defense against infections due to their ability to kill without histocompatibility complex recognition but has been observed to be reduced following burn injury (6, 53, 54). In the present study, we observed lower median levels of CD4+ lymphocytes as well as NK cells in individuals who developed NI relative to No NI and HCs (**Figures 2A, C**).

In addition to number of T cells we also examined differences in T-cell function as a measurement of cytokine production after PHA stimulation. PHA binds and crosslinks the surface proteins, including the T cell receptor, which induces T cell activation. Similar to LPS stimulation assays, PHA has been well validated and been used to predict poor outcomes in various populations of several disease states, including pediatric burns (12, 55–60). In the present study we show that burn injury, regardless of infection, resulted in lowered cytokine production following PHA stimulation for all the tested cytokines (**Figures 3B–D**). However, only PHA-induced IL-10 production capacity was significantly different in burn subjects who developed NI as compared to No NI. Therefore, while unstimulated plasma IL-10 is higher in patients who develop NI, it appears not to be a result of lymphocyte production of IL-10, further connecting the dysregulation of the immune cells after thermal injury that resulted in infectious complications.

Tregs are a subset of CD4+ T cells and generally have strong immune suppression activity and downregulate CD4+ T cells and NK cells (61). Numerous studies of sepsis in adults have shown increases in percent Tregs and Treg activity (as measured through cytokine expression of IL-10 and TGF-β and as expression of CTLA-4 and FOXP3 on the cell surface) and implicate Tregs in adaptive immune suppression (62–65). Still others have shown absolute counts of Tregs have been decreased in patients with infections (62, 66). Therefore, the likely increase in percent Tregs is caused by the loss of CD4+ T cells rather than the increase in absolute Tregs, but this has not been explored in pediatric burn injury. In our work the absolute Tregs were found to be significantly lower in burn patients who would develop NI compared to No NI and HCs (**Figure 2B**), but there was no difference in percent Treg expression (data not shown). This is a novel finding and suggests immune dysfunction in this cohort may be a result of alternative T cell inhibitory pathways.

To explore this, we examined two of the most well-studied inhibitory checkpoint regulators that play a crucial role in the regulation of T cells: PD-1 and CTLA-4. Both PD-1 and CTLA-4 are receptors expressed on several cell types including CD4+ lymphocytes, NK cells, and Tregs (67, 68). Both receptors have been well-validated, in human sepsis studies, to be upregulated on various immune cells, which leads to a dysregulated T cell response and subsequent immunosuppression (69, 70). Similarly, burn murine models have exhibited increased PD-1, but this research is still relatively limited in terms of human thermal injuries and is non-existent in pediatrics (65, 71, 72). Within the present study, percent PD-1 expression on CD4+ lymphocytes was elevated for burn patients within the first 3 days of injury relative to HC (**Figure 2G**). Percent CTLA-4 on CD4+ lymphocytes was also slightly elevated after burn injury although not statistically significant (**Figure 2H**). However, there was no difference for PD-1 or CTLA-4 expression between those who went on to develop a NI and those who did not. Therefore, while these proteins likely contribute to initial immune suppression in burn injury, other proteins could be compromising the immune system that ultimately leads to infection.

To this end, we studied several soluble proteins found in the unstimulated plasma that play a key role in the regulation of T cells. Similar to the flow cytometry results above, neither soluble CTLA-4 nor soluble PD-1 were significantly different between burn groups (**Supplementary Figure 1**). However, we did observe a significant increase in other soluble T cell inhibitory proteins: BTLA and TIM-3 (**Figures 4A, B**). BTLA is expressed, as the name suggests, on B and T lymphocytes, but also on macrophages, monocytes, NK cells, and dendritic cells, while TIM-3 is constitutively expressed on innate immune cells (73–75). Some research has observed that soluble BTLA and TIM-3, like PD-1 and CTLA-4, is elevated in sepsis patients (74, 76–80). Unfortunately, both soluble TIM-3 and BTLA have received less attention as immune checkpoints during injury that results in infections with no human studies exploring their role in burn injury. Remarkably, soluble CD27 is also significantly increased in burn patients who develop infections relative to those who did not (**Figure 4C**). Unlike BTLA and TIM-3, plasma levels of soluble CD27 are typically used as a marker of immune activation and currently has not been studied for its role in infection (i.e., sepsis) or burn injuries. However, Agematsu et al. (1994), found that soluble CD27 inhibits T cell proliferation, indicating that the increased soluble CD27 may also relate to the decreased T cells observed within our study population (81). Thus, soluble BTLA and TIM-3 as well as CD27 represent intriguing proteins for further research in burn injury as these proteins may be playing a key role in augmenting an appropriate immune cell response, leaving these individuals highly susceptible to infections. Further these proteins could hold promise as targets of immunomodulatory therapies.

We next wanted to observe ROCs to assess the ability of these immune markers and soluble proteins to predict the subsequent development of NI. We found that the highest AUC was LPS-induced TNFα production capacity (0.82; **Figure 5**), which has been repeatedly shown in many different patient populations to be a highly predictive marker of infection (7, 26, 27, 45). Interestingly, the next highest AUCs were for the soluble proteins CD27, BTLA, and TIM-3 (0.81, 0.78, and 0.77, respectively) and unstimulated plasma IL-10 (0.78) (**Figure 5**). This further confirms the relationship observed for those that develop infections after burn injury relative to those that do not. Likely, the elevated plasma IL-10 (optimal threshold >11.59 pg/mL) inhibits antigen presentation, decreasing ex vivo LPS-stimulated TNF-α production capacity (optimal threshold < 505.8 pg/mL) leaving the individual highly susceptible to infections by decreasing T cell immunity (7, 27, 47, 49, 50). While there is still limited research on soluble CD27, BTLA, and TIM-3, these markers represent highly important indicators (optimal threshold >1129 pg/mL, >1353 pg/mL, and >705.3 pg/mL, respectively) for the development of infection in pediatric burn injury. Through further investigation, these soluble proteins may bridge the gap between the mechanisms underlying these forms of immune suppression following burn injury. This discovery is important because increasing evidence suggests that critical illness and injury-induced immune suppression is reversible using immunostimulatory therapies (82, 83). Therefore, immunomodulators targeted at these proteins could be very promising to restore immune function in this vulnerable and underrepresented population. Moreover, these soluble biomarkers, in conjunction with LPS-induced TNFα, could be used as a standard method to identify those at risk for infection after burn injury.

There are several limitations in this study to note. First, this study was performed at a single center, made to ensure standardization and reproducibility of the tests, but could limit the extrapolation of our results to other centers. Moreover, we had a limited sample size especially for those that developed NI. As a result, we were limited in the number of covariables that could be tested, and it is possible that other confounding variables not tested may affect the results as well as its influence of immune function (e.g., number of procedures, transfusions and use of medication such as opioids). Additionally, while quite a few markers were tested, this was not an exhaustive panel. We cannot therefore comment on other elements of innate immune function such as migration, phagocytosis, and intracellular killing or adaptive immunity such as CD8 cells and B-cell activation. Another limitation is that this analysis was conducted using many statistical tests and these tests represent only exploratory findings. Moreover, while Dunn’s multiple comparison test and Bonferroni’s correction were used for the pairwise comparisons made between three groups, the rest of the tests were not adjusted for multiple testing. We also explored only the comparative performances of individual immune markers on the prediction of infection but were unable to explore the best combination of markers to predict NI. Therefore, future work with increased sample size or multi-center analysis would allow us to adjust for potential confounding factors (e.g., burn wound size and depth, mechanism of injury) and perform more exploratory analysis on the combination of markers in predicting NI. Despite this limitation, we still demonstrate a significant link between suppressed immune function, elevated soluble protein markers, and the development of NI after burn injury.

In conclusion, we have found evidence of increased soluble BTLA and TIM-3 as well as CD27 in the immunosuppressed pediatric burn patients who go on to develop infections. This research further advances the knowledge of suppressed immune function in pediatric burn patients. Importantly these soluble protein markers, which were measured in the first 3 days after injury, were highly predictive of patients who would develop infectious complications (median time to diagnosis was 8 days). Therefore, these findings represent an intriguing opportunity to apply targeted immunomodulatory therapies for these proteins before the onset of infection to perhaps decrease the rate of this devasting complication. Additional work is needed to confirm these findings and to further understand the mechanisms driving this form of immune dysfunction in pediatric patients with thermal injury.

## Data availability statement

The raw data supporting the conclusions of this article will be made available by the authors, without undue reservation.

## Ethics statement

The studies involving human participants were reviewed and approved by Institutional Review Board of Nationwide Children’s Hospital. Written informed consent to participate in this study was provided by the participants’ legal guardian/next of kin.

## Author contributions

MH and RT conceived the study. AW, JB, and JH recruited patients and collected samples and subject data. RF and RT were responsible for patient care and interpretation of clinical data. JP and SS performed experiments. JP, RA, and RT were involved in data analysis. JP and RT wrote the manuscript. RA, SS, AW, JB, JH, RF, and MH assisted on writing and revising of the manuscript. All authors contributed to the article and approved the submitted version.

## Funding

Research reported in this publication was supported by the National Institute of General Medical Sciences (K08GM124499) and National Institute of Child Health and Human Development (K12HD047349) of the National Institute of Health.

## Acknowledgments

We are grateful for the work of all involved physicians, surgeons and nurses of the burn center at Nationwide Children’s Hospital.

## Conflict of interest

The authors declare that the research was conducted in the absence of any commercial or financial relationships that could be construed as a potential conflict of interest.

## Publisher’s note

All claims expressed in this article are solely those of the authors and do not necessarily represent those of their affiliated organizations, or those of the publisher, the editors and the reviewers. Any product that may be evaluated in this article, or claim that may be made by its manufacturer, is not guaranteed or endorsed by the publisher.
