# Supplementary material for: Early detection of soluble CD27, BTLA, and TIM-3 predicts the development of nosocomial infection in pediatric burn patients
Source: Front Immunol. 2022 Jul 26;13:940835. doi: 10.3389/fimmu.2022.940835 (PMC9360547; doi:10.3389/fimmu.2022.940835)
Supplement: Supplementary file 1 [file DataSheet_1.docx]

Supplementary Material

# Supplementary Figure and Table

## Supplementary Figure

**Supplemental Figure 1. Soluble protein in unstimulated plasma within the first 72 hours following pediatric thermal injury.** Soluble CD152, CD137, CD28, CD80, IDO, LAG-3, PD-1, PD-L2 (A-H), measured in the unstimulated plasma, displayed no statistically significant differences in those that developed an infection (n=22) relative to those who did not (n=59) or healthy controls (n=12). Statistical analysis was performed using one-way ANOVA plus Dunn’s test. Lines represent median and interquartile range; dots represent the individual data points. NI, nosocomial infection (red); No NI, no infection (blue); HC, healthy controls (green)

## Supplementary Table

**Supplemental Table 1. Receiver operating characteristics curve analysis**

| **Biomarker** | **AUC** | **p-value** | **Youden's Index** |
| --- | --- | --- | --- |
| CD4 Regulatory T Cells | 0.6976 | 0.0096 | < 37.00 cells/μL |
| Plasma IL-8 | 0.7038 | 0.0041 | > 189.2 pg/mL |
| CD14+ Monocytes | 0.7138 | 0.0051 | < 701.0 cells/μL |
| %HLA-DR Expression | 0.7215 | 0.0038 | < 81.50 cells/μL |
| PHA-induced IL-10 | 0.722 | 0.0016 | < 106.6 pg/mL |
| CD4+ Lymphocytes | 0.7292 | 0.0026 | < 532.0 cells/μL |
| Plasma IL-6 | 0.7324 | 0.0011 | > 49.41 pg/mL |

AUC: area under the curve
